# Effects of Frozen Red Dragon Fruit Consumption on Metabolic Markers in Healthy Subjects and Individuals at Risk of Type 2 Diabetes

**DOI:** 10.3390/nu17030441

**Published:** 2025-01-25

**Authors:** Mildred Inna Marcela Flores-Verastegui, Shelly Coe, Jonathan Tammam, Haythim Almahjoubi, Robyn Bridle, Sabina Bi, Pariyarath Sangeetha Thondre

**Affiliations:** Oxford Brookes Centre for Nutrition and Health, School of Sport, Nutrition and Allied Health Professions, Oxford Brookes University, Oxford OX3 0BP, UK or inna_fv@hotmail.com (M.I.M.F.-V.); scoe@brookes.ac.uk (S.C.); jtammam@brookes.ac.uk (J.T.);

**Keywords:** red dragon fruit, blood pressure, blood glucose, glycaemic response, insulinaemic response

## Abstract

Background/Objectives: The interest in creating new products to decrease the risk of developing non-communicable chronic diseases such as type 2 diabetes (T2D) is increasing. These products include traditional food sources used as part of diverse cultures around the world, such as dragon fruit. The aim of this study was to investigate the effects of a frozen red dragon fruit (FRDF) beverage on blood pressure, glycaemic response (GR) and insulinaemic response (IR), lipid profile (LP), total antioxidant status (TAS), and C-reactive protein (CRP) levels in healthy subjects and individuals at risk of T2D. Methods: A parallel design trial (UREC registration number 211527; ClinicalTrials.gov registration number NCT05199636/19 January 2022) lasting four weeks and involving three testing sessions was conducted; participants were randomly assigned to one of two treatments (following general health guidance or consuming FRDF beverage). Systolic and diastolic blood pressures were taken; venous blood samples were collected to determine the LP and CRP levels; and capillary blood samples were taken before and after consuming a standard glucose drink to evaluate GR and IR at 15 min intervals (first hour) and 30 min intervals (second hour). Results: Eighteen participants completed this study, nine healthy (28.44 ± 5.20 years) and nine at risk (31.78 ± 12.11 years). The daily consumption of an FRDF-based beverage for four weeks by individuals at risk of T2D resulted in a reduction in blood pressure and IR–incremental area under the curve. The LP showed a downward trend, and a significant difference between treatments (*p* = 0.009) was found for CRP levels. Conclusions: Beverages based on FRDF may have the potential to decrease the risk of T2D.

## 1. Introduction

According to the World Health Organization, more than 420 million people worldwide have diabetes. Diabetes is defined as a chronic condition linked to abnormally high levels of glucose in the bloodstream. There are two main types of diabetes: insulin-dependent diabetes also known as type 1 (T1D) and non-insulin-dependent diabetes or type 2 (T2D) [1]. T2D is a condition that has been linked to lifestyle, which includes eating habits and physical activity, in addition to hereditary factors [2,3]. A family history of T2D, being overweight, and age are some of the other main factors that can put a person at risk of developing this type of diabetes. Moreover, a sedentary lifestyle, sleeping disorders, and other conditions such as high blood pressure are also considered as risk factors for T2D [4,5,6].

Fasting plasma glucose (FPG) levels, the oral glucose tolerance test (OGTT), and glycated haemoglobin (HbA1c) quantification can be used to determine whether someone is at risk of developing T2D. When FPG levels are higher than 7 mmol/L for two separate tests, the WHO’s Global Health Observatory states that this is a diagnosis for T2D [7]. Normal FPG values are between 3.9 and 5.6 mmol/L, and people with higher plasma levels than normal but lower than diabetes levels are considered at risk of developing T2D [8].

The interest in creating new food products to help reduce the risks of developing chronic diseases such as T2D has been rising. Most of these products have been based on natural food and drink sources that have been used in a traditional way to manage conditions by different cultures over time, for example, tropical fruits rich in bioactive compounds such as watermelon and guava [9]. Dragon fruit (*Hylocereus* sp.), a tropical fruit containing between 10 and 12% total sugar, including glucose, fructose, and xylose [10], has been shown to have a high content of bioactive compounds including polyphenols that are linked to positive health outcomes in conditions such as T2D and hypertension [11]. Thus, clinical trials to investigate the effects of dragon fruit in individuals at risk of these health conditions are warranted.

The effects of red flesh dragon fruit (*Hylocereus polyrhizus*) on health have been studied in a small number of clinical trials. Fresh red dragon fruit has been tested in participants who are overweight/obese and in individuals with T2D as part of seven-day and ten-day trials, respectively [12,13], showing a reduction in blood glucose levels. The effects of consuming red dragon fruit juice for 14 days were investigated in women with high total cholesterol levels and in subjects with T2D [14,15]; the findings demonstrated a reduction in cholesterol and glucose levels, respectively. Powder obtained from dried red dragon fruit was investigated in both healthy individuals in a 14-day trial [16] and individuals at risk of T2D in a four-week study [17]; the results exhibited improved endothelial function for the former and a reduction in plasma glucose levels for the latter. One further clinical trial was conducted in participants with T2D for a period of four weeks, without stopping their medication, showing a significant decrease in blood glucose and triglyceride levels [18]. Positive findings have been linked to the presence of bioactive compounds, such as polyphenols in fresh and dehydrated red dragon fruit; however, the effects of its consumption have not yet been compared to the effects of following healthy eating guidance.

The main objective of this short-term study was to investigate the effects of a frozen red flesh dragon fruit (FRDF)-based beverage on blood pressure, blood glucose, glycaemic response (GR), and insulinaemic response (IR), after four weeks of consumption using a parallel study design in healthy subjects and those at risk of T2D. The secondary aim was to determine the effect of this FRDF-based beverage on cholesterol levels, triglyceride levels, total antioxidant status (TAS), and C-reactive protein (CRP) levels.

## 2. Materials and Methods

### 2.1. Study Design and Setting

A parallel study was designed, in which the effects of two treatments (following general health guidelines and consuming FRDF-based beverage) on blood pressure, blood glucose, insulin response, and biomarkers were investigated in individuals at risk of T2D and in healthy individuals at Oxford Brookes Centre for Nutrition and Health (OxBCNH). Each participant was randomly assigned to one testing group using Research Randomizer (https://www.random.org, accessed on 22 August 2021). Participants and researchers were not blinded because the comparison was between the effects of consuming an FRDF-based beverage in the intervention group and adherence to general healthy eating guidelines in the control group, and the control group was not required to consume a placebo beverage. A sample size of 32 participants was calculated to have 90% power at the 0.05 level of significance (two-sided) to detect a difference of 1.0 ± 0.8 mmol/L for FPG (*p* = 0006) in individuals at risk of T2D. This calculation was based on a previous study evaluating individuals with prediabetes, where the difference in FPG levels between those consuming 100 g of fruit and those in the control group was 1 mmol/L after 4 weeks of consumption [17,19]. In the above-mentioned human study, 36 participants were split into 18 participants per group and again divided into 3 groups of 6 participants each, to receive three different treatments [17]. This study lasted four weeks and involved three testing sessions. Participants were asked either to consume 330 mL of an FRDF-based beverage once a day for the duration of the study period or to follow general health guidelines for four weeks. This study with the registration number UREC 211527 was approved by the University Research Ethics Committee and was registered on ClinicalTrials.gov with the identifier NCT05199636 in January 2022.

### 2.2. Participants

Participants between 18 and 65 years old were recruited by posters on Oxford Brookes University and Oxfordshire notice boards including those in groceries stores and community centres and an advertisement posted in Daily Info.

Interested volunteers received two participant information sheets by email, one for individuals at risk of T2D and the other for healthy individuals. If potential participants agreed to take part and were deemed eligible, three sessions were arranged to visit the OxBCNH, located at the Headington Campus, Oxford.

Volunteers who met at least two of the following criteria were selected to take part in this study as individuals at risk of T2D: [a] have a parent or sibling with T2D, [b] have a body mass index (BMI) greater than or equal to 25.0 kg/m^2^, or [c] have a sedentary lifestyle (low/moderate physical activity). Volunteers with a BMI between 18.5 and 24.9 kg/m^2^ and without diabetes, hypertension, or kidney disease were eligible as healthy participants. Exclusion criteria included COVID-19-related symptoms, allergies to dragon fruit, taking medication that requires a prescription (not including supplements or other over-the-counter medication such as pain killers), being pregnant, or breastfeeding.

### 2.3. Treatments

#### 2.3.1. Group 1

Group 1 received advice to follow general health guidelines based on the Eatwell Guide [20] daily for four weeks and received guidelines for T2D prevention from the National Institute for Health and Care Excellence (NICE) [21]. As supporting materials, an Eatwell Guide and a treatment follow-up log were given to participants to monitor whether the participants followed the advice.

#### 2.3.2. Group 2

The treatment for group 2 involved drinking 330 mL of an FRDF-based beverage daily for four weeks. The components of the product were 217 g of frozen dragon fruit pulp (My Exotic Fruit, Ingatestone, UK), 80 mL of water, and 20 mL of lime juice (Tesco Plc, Welwyn Garden City, England, UK). To prepare the beverage with a concentration of polyphenols of 303.04 μg GAE/mL, dragon fruit pulp was frozen at −18 °C for a week to be ground in a blender (Nutribullet 600 series) for one minute; then, water and lime juice were added and stirred before consumption. At the beginning of the intervention, the product was made by a researcher; after that, the beverage was prepared by the participants following a standard procedure provided by the researcher, using the individual portion sizes of the frozen fruit, as well as the measuring cups given to accurately measure the quantity of water and lime juice. Frozen fruit, lime juice, and the standard procedure were given to the participants to take away after the first and second sessions to prepare the treatments at home. Paper logs for their treatment follow up as well as comments or side effects were recorded daily by the participants in the intervention group.

### 2.4. Protocol

**Figure 1:**
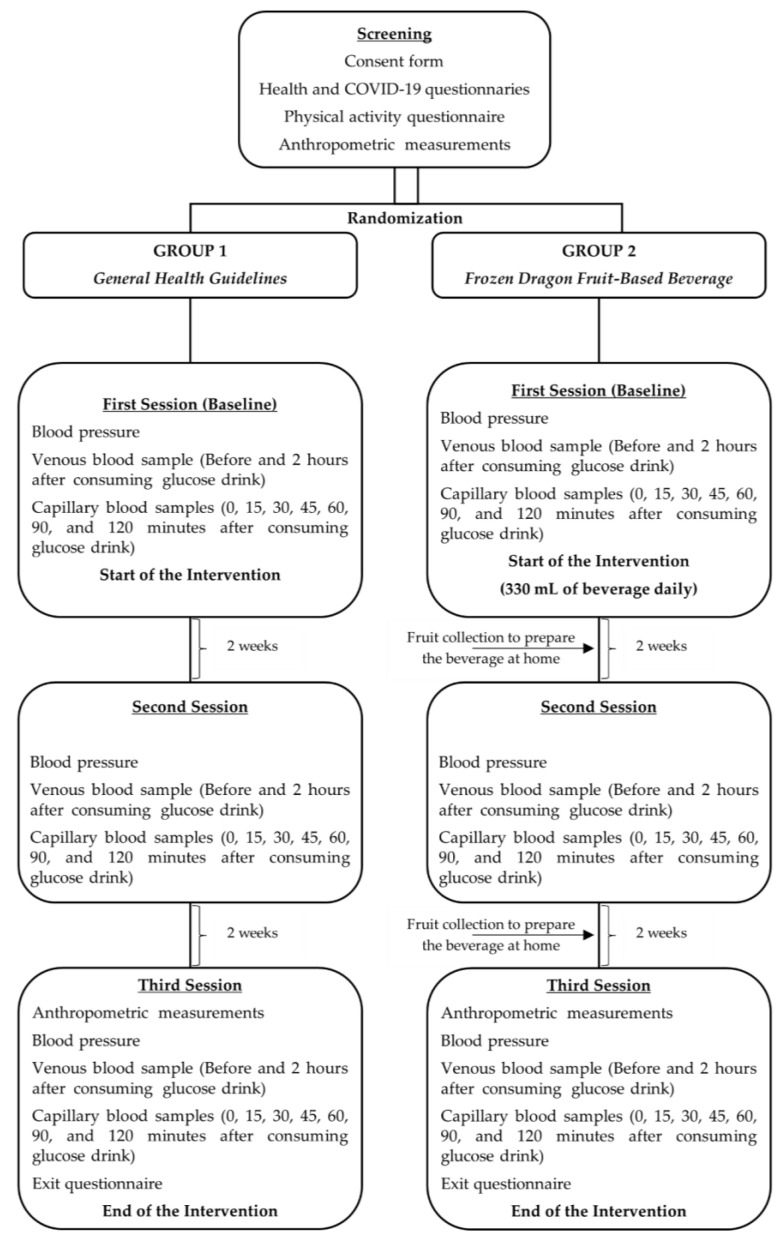
Flow chart of study protocol.

Figure 1 shows a flow chart from screening to the end of the intervention.

#### 2.4.1. First Visit

- Screening: In a fasted state, screening was carried out in the OxBCNH consulting room. Each volunteer provided written consent and completed two screening questionnaires (screening for general health and COVID-19) and the International Physical Activity Questionnaire (IPAQ). Participants were given the option to give both venous and finger prick blood samples or just finger prick blood samples. BMI data were collected; height was measured barefoot using a Seca274 free-standing digital stadiometer (Seca LTD, Birmingham, UK); and weight was measured using an MC-980MA plus body composition analyser (Tanita, Amsterdam, The Netherlands).
- Testing session: Systolic and diastolic blood pressure was taken after 10 min of rest with a UA-767 plus digital blood pressure monitor (A&D Medical, Tokyo, Japan); two more measurements were taken at five-minute intervals. From those who agreed, 9 mL of venous blood sample was collected via venepuncture from fasted participants with a BD-Vacutainer Eclipse blood collection needle and holder and placed into serum separation tubes (BD Vacutainer SST Advance) to determine TAS and cholesterol, triglyceride, and CRP levels. Baseline capillary blood samples were taken by finger pricking using a Unistick-3 lancet and analysed on the Glucose 201 DM system. Following this, a standard [22] glucose drink [75 g of glucose (Myvegan, Myprotein, Altrincham, England, UK) in 250 mL of water] was consumed by participants to determine glucose tolerance for the oral glucose tolerance test (OGTT), and a 4 mL venous sample was taken after two hours. During the two hours, finger prick blood samples were taken with a single-use Unistick-3 lancet (Owen Mumford, Woodstock, UK) at 0, 15, 30, 45, 60, 90, and 120 min intervals to avoid taking too many venous blood draws in two hours. These samples were collected to evaluate the glycaemic and insulinaemic response profiles, using a Glucose 201 DM System blood glucose analyser (HemoCue, Ängelholm, Sweden) and a Cobas e411 electro-chemi-luminescence immunoassay analyser (Roche Diagnostics, Burgess Hill, UK), respectively. The treatment described in Section 2.3 was given to the participants to start the intervention.

#### 2.4.2. Second Visit

The second testing session was held two weeks after the treatment began, and the protocol described in Section 2.4.1 for the first testing session was followed.

#### 2.4.3. Third Visit

After four weeks (±1 day) of treatment, the third session was conducted, as mentioned in Section 2.4.1. Anthropometric measurements were recorded, and an exit questionnaire was provided. The participants were asked to return the follow-up log or consumption log as a hardcopy record to the researcher.

### 2.5. Blood Analysis

#### 2.5.1. Glycaemic Response (GR)

Capillary blood samples obtained via a finger prick with a single-use Unistick-3 lancet (Owen Mumford, UK) were collected, after discarding the first two drops, using a 5 μL disposable micro-cuvette (HemoCue, Sweden). These were analysed immediately in the Glucose 201 DM System (HemoCue, Sweden) to record the blood glucose level in mmol/L. A calibration test was performed prior to each testing session with the GlucoTrol-NG, Level 2 (HemoCue, Sweden).

#### 2.5.2. Insulinaemic Response (IR)

After the capillary blood samples for GR were taken, a pre-chilled microtube containing dipotassium ethylendiaminetetraacetic acid (K_2_EDTA) (BD Microtainer, London, UK) was used to collect approximately 375 μL of blood samples for insulin analysis. Samples were placed immediately on ice until centrifugation at 4000 rpm for 10 min (Sigma 1–14, Catcliffe, UK) to separate the plasma which was transferred into 1.5 mL Eppendorf tubes and stored at −20 °C until analysis was conducted in a Cobas e411 automated analyser (Roche Diagnostics, UK).

#### 2.5.3. Biomarkers

Venous blood samples collected in serum separation tubes (BD Vacutainer SST Advance) were centrifuged in a Biofuge Primo (Heraeus Instruments, Brentwood, UK) at 1300× *g* for 10 min. Serum was separated and placed into 1.5 mL labelled Eppendorf tubes and transferred into a freezer at −20 °C until photometric analysis was conducted using a Daytona Plus fully automated analyser (Randox, London, UK) for cholesterol levels, triglyceride levels, TAS, and CRP levels.

### 2.6. Statistical Analysis

SPSS, version 28, was used to conduct the statistical analysis of the data following the intention-to-treat concept. The incremental area under the curve (iAUC) values for GR and IR for healthy individuals and individuals at risk of T2D were calculated using the trapezoidal rule [23]. The normality of the data was tested prior to the statistical analysis using a Shapiro–Wilk test; a natural log transformation was performed for data with a non-normal distribution. The homogeneity of variance assumption was checked using Levene’s test. Sphericity was determined by Mauchly’s test, and when the assumption was not met, a Greenhouse–Geisser adjustment was made. A mixed ANOVA was used to examine interactions between three time points (“week-0”, “week-2”, and “week-4”, within the participants’ groups) and treatments (following “general health advice” and consuming “FRDF-based beverage”, between participants’ groups) for all variables. A repeated measures ANOVA with post hoc analysis with a Bonferroni correction was used to determine differences between time points for all variables within each treatment group. The results are shown as the mean ± SD with significance accepted at the alpha level of *p* < 0.05.

## 3. Results

**Figure 2:**
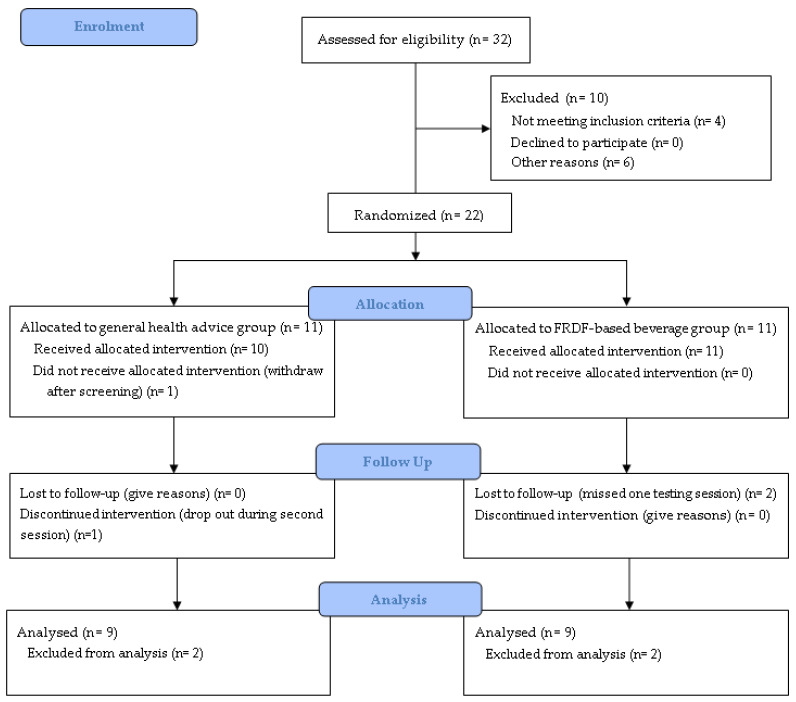
Consort flow diagram.

Thirty-two volunteers aged between 16 and 60 years old were recruited to participate in the screening session. Four volunteers did not fulfil the inclusion criteria, four more did not arrive due to personal circumstances, one was taking blood pressure medication, and one was unable to fast. Therefore, twenty-two participants consented to start this study, one dropped out after the first testing session, another one dropped out during the second session, and two more missed one testing session (Figure 2). Eighteen participants (twelve females and six males) completed this study, nine healthy and nine at risk of T2D, as Table 1 shows.

The participants at risk of T2D belonged to five different ethnicities (Asian, Black, Mixed, White, and Other), while the healthy participants were either of Asian or White ethnicities. A significant difference was found for weight (*p* = 0.009) and BMI (*p* = 0.004) between healthy participants and those at risk of T2D. There was no significant interaction between time and treatment for weight (*p* = 0.48) and BMI (*p* = 0.082). Weight and BMI exhibited no significant difference between pre- and post-intervention at *p* = 0.386 and *p* = 0.164, respectively. (Table 1).

### 3.1. Blood Glucose

Table 2 shows the fasting blood glucose and blood glucose concentration after two hours of drinking a standard glucose solution in groups receiving the general health advice or the FRDF beverage at three time points.

There was no significant difference between time points for subjects at risk of T2D for fasting blood glucose, *p* = 0.745, and there was no evidence of a main effect of treatments, *p* = 0.296. Interaction between time and treatment was not significant, *p* = 0.911.

Fasting blood glucose levels for healthy participants exhibited a non-significant difference between time points, *p* = 0.437, and a significant difference between treatments, *p* = 0.036. There was no interaction between time and treatment, *p* = 0.771.

Glucose levels after two hours did not show a significant difference between time points, *p* = 0.521, for individuals at risk of T2D. There was a significant difference between treatments, *p* = 0.038, and no significant interaction between time and treatment *p* = 0.151.

No difference between time or treatments was found for healthy participants, and there was no interaction between them at *p* = 0.331, *p* = 0.433, and *p* = 0.193, respectively.

### 3.2. Glycaemic Response During the Oral Glucose Tolerance Test

**Figure 3:**
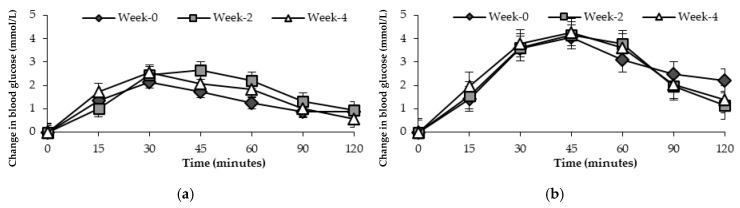
Mean changes in blood glucose response (mmol/L) over 120 min after consuming standard glucose drink in participants at risk of T2D at three time points. (**a**) General health advice; (**b**) frozen red dragon fruit-based beverage.

**Figure 4:**
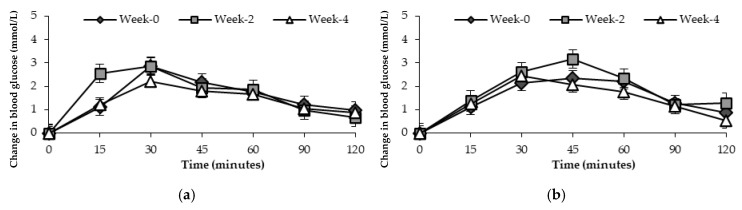
Mean changes in blood glucose response (mmol/L) over 120 min after consuming standard glucose drink in healthy participants at three time points. (**a**) General health advice; (**b**) frozen red dragon fruit-based beverage.

Changes in blood glucose over two hours after the consumption of a standard glucose drink in groups receiving general health advice or the FRDF beverage at three time points are shown in Figure 3 and Figure 4.

Blood glucose values for participants at risk of T2D showed no significant difference over time, *p* = 0.420 for participants drinking the FRDF beverage, and *p* = 0.143 for those receiving general health advice after a Greenhouse–Geisser correction (Figure 3)

No significant difference was found between the three time points evaluated for blood glucose in healthy participants following general health advice or receiving the FRDF beverage at *p* = 0.833 and *p* = 0.143, respectively (Figure 4).

Table 3 shows the GR-iAUC results. There was no significant effect of time points on the GR-iAUC in participants at risk of T2D at *p* = 0.834; however, a significant main effect of treatments was found at *p* = 0.021. No significant interactions between time and treatments were found at *p* = 0.644. No significant effect of time, *p* = 0.714, or between treatments, *p* = 0.918, was found in healthy participants. There were no significant interactions between time and treatments, *p* = 0.746.

### 3.3. Insulinaemic Response During the Oral Glucose Tolerance Test

**Figure 5:**
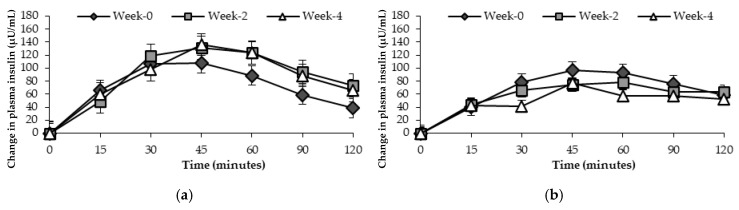
Mean changes in plasma insulin response (μU/mL) over 120 min in participants at risk of T2D at three time points. (**a**) General health advice; (**b**) frozen red dragon fruit-based beverage.

Figure 5 shows the change in plasma insulin response in participants at risk of T2D. There was evidence of a main effect of time points (week-0, week-2, week-4) on insulin concentration after the consumption of a standard glucose drink in participants at risk of T2D receiving general health advice, *p* = 0.043. The change in insulin response in those at risk of T2D who consumed the FRDF-based beverage was not significant between times, *p* = 0.063.

**Figure 6:**
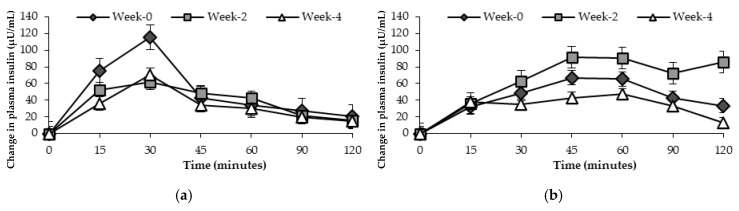
Mean changes in plasma insulin response (μU/mL) over 120 min in healthy participants at three time points. (**a**) General health advice; (**b**) frozen red dragon fruit-based beverage.

Changes in plasma insulin response in healthy participants are presented in Figure 6. There was no effect of time points (week-0, week-2, and week-4) on insulin concentration after the consumption of the standard glucose drink in healthy participants after Greenhouse–Geisser correction, *p* = 0.705 for those receiving general health advice, and *p* = 0.080 for those who consumed the FRDF-based beverage.

The IR-iAUC results are shown in Table 4. After conducting a repeated measures ANOVA with a Greenhouse–Geisser correction, the IR-iAUC results for participants at risk of T2D were found to be non-significant across the three time points, *p* = 0.299, and there was a non-significant difference between treatments, *p* = 0.497. Interactions were not significant, *p* = 0.514.

Healthy participants showed no significant main effects of time points, *p* = 0.250. Between the group that received general health advice and the group that consumed the FRDF-based beverage, no significant effect of treatment was found, *p* = 0.704. There was also no significant interaction between time and treatments at *p* = 0.463.

### 3.4. Blood Pressure

The systolic and diastolic blood pressure results are shown in Table 5. There was no evidence of a main effect of time points on treatments, *p* = 0.886, for systolic blood pressure evaluated in the participants at risk of T2D, and there was no significant effect of treatments, *p* = 0.808. The interaction between time and treatment was not significant at *p* = 0.719. Systolic blood pressure for healthy participants showed no significant difference in time, *p* = 0.169, and between treatment, *p* = 0.695. There was no significant interaction between time and treatment at *p* = 0.695.

There was a numerical reduction in diastolic blood pressure in those at risk of T2D after four weeks of treatment; however, there was no significant difference at *p* = 0.396. The difference between treatment was not significant, *p* = 0.831, and there was no significant interaction between three time points and treatment, *p* = 0.766. No significant difference between three time points was found for diastolic pressure in healthy participants, *p* = 0.565. Treatment differences were not significant, *p* = 0.701, and there were no interactions between time and treatments, *p* = 0.857.

### 3.5. Biomarkers

Table 6 shows values for cholesterol, triglycerides, TAS, and CRP in serum obtained from seven participants as eleven participants refrained from giving venous blood samples. Table 6 only includes biomarker values for healthy individuals who received general health advice as none of the healthy participants that consumed the FRDF-based beverage gave venous blood samples.

After conducting a mixed ANOVA, the results showed that there was no significant difference between time points for cholesterol, triglycerides, and TAS in participants at risk of T2D, *p* = 0.539, *p* = 0.907, and *p* = 0.369 respectively. There was evidence of the main effects of treatments on CRP levels, *p* = 0.009. No significant difference was found in healthy participants between time points.

## 4. Discussion

The aim of this study was to determine the effects of consuming an FRDF beverage during a four-week period in healthy individuals and individuals at risk of T2D, and to our knowledge, this is the first study to explore the health effects of consuming a frozen form of red flesh dragon fruit. A non-significant decrease in blood pressure, IR-iAUC, cholesterol, and triglycerides and a significantly reduced level of CRP were found in individuals at risk of T2D after a four-week intervention in those consuming the FRDF-based beverage when compared to those receiving general health advice. Healthy subjects showed a decrease in IR-iAUC regardless of the treatment received. The ethnicities of the participants at risk included those mentioned by the National Health Service as risk factors for developing T2D, such as Asian and Black origin [4]. Although the duration of this study is not sufficient to show clinically relevant changes in healthy participants in the outcomes measured, the results may provide mechanistic information on the effects of dietary components, which can be useful in providing dietary recommendations and preventing chronic diseases such as type 2 diabetes [24].

Research into polyphenol compounds has increased in the last decade, and this is likely due to their presence in a variety of foods globally and the current interest in creating novel foods associated with health benefits [25]. Studies suggest that the antioxidant properties of polyphenol-rich foods are responsible for their role in glucose metabolism: the rise in insulin secretion, the promotion of beta cell proliferation, and the inhibition of intestinal enzymes have been attributed to some polyphenols [26,27]. Clinical trials have been carried out in which the effects of consuming red flesh dragon fruit on glucose metabolism were investigated in those with a BMI higher than 25.0 kg/m^2^ and those with T2D. In a study where thirty-two people who were overweight/obese were selected to eat 180 g per day of red dragon fruit flesh for seven days, the results showed a non-significant difference between fasting blood glucose levels at baseline and those after the intervention [12]. Similar results were found in this study as there was no difference in fasting blood glucose levels between time points in healthy individuals and those at risk of T2D, who consumed 330 mL of an FRDF beverage (an increased dose of dragon fruit by 17%). Studies carried out to investigate other high-polyphenol fruits such as strawberries and cranberries have found similar results in terms of fasting glucose levels in participants with metabolic syndrome [28,29]. However, interventions investigating the effects of red dragon fruit consumption in participants with T2D found a significant difference in blood glucose levels between baseline and those after the treatment. Girsang et al. [15] found a significant decrease in blood glucose levels from 19.9 to 18.0 mmol/L in 30 subjects with T2D after a 14-day consumption of 250 mL of red dragon fruit juice; however, the results should be handled with caution as they are presented as the difference observed before and after the consumption of the product, and the study design did not consider a control group. The findings from a four-week trial evaluating the effects of the consumption of 400 g per day of dragon fruit by 22 individuals with T2D who were taking medication to control glucose levels showed a significant reduction of 24.02% in fasting blood glucose levels after a four-week treatment [18]. This effect could probably be due to the amount of dragon fruit consumed (84% more than that in the current study) in addition to the medication intake; the researchers attributed their results to the quantity of bioactive compounds, such as polyphenols, and also vitamin C and fibre. Antioxidants have been linked to a reduction in blood glucose levels increasing the secretion of insulin [30] and reducing oxidative stress [15,31]; meanwhile, dietary fibre has been reported to have a positive impact on reducing blood glucose levels due to its capacity to absorb water and reduce digestion and gastric emptying, therefore slowing the absorption of glucose [12,18,32]. Polyphenol-rich diets have been reported to have effects on reducing the presence of glucose in the blood [33] via diverse mechanisms such as the inhibition of glucose absorption [34] or stimulating insulin secretion [35].

Discrepancies between the results from the studies mentioned above and those found in this study may be due to the form and amount of fruit provided to the participants; the variability in polyphenol content, which was not reported in the previous studies; the experimental design; the sample size; and the condition of the participants. In this study, the frozen from of dragon fruit was investigated, suggesting that the food matrix within which the polyphenols are held may influence their bioactive properties; although freezing is known to preserve the antioxidant compounds in whole fruits, any damage to the cell structure during storage, thawing, or preparation may contribute to their leaching and oxidation [36]. The concentration of polyphenols was 303.04 μg GAE/mL, and a sample size of 18 individuals (healthy and at risk of T2D) was assessed in a parallel design. Polyphenols have shown to reduce the blood glucose levels in T2D subjects [37], where it is easier to detect changes due to more imbalanced glucose regulation. Meanwhile, in the cohorts evaluated in the current study, it is more difficult to observe a reduction in blood glucose than in those with T2D due to the plausible homeostatic mechanisms in the human body.

As far as we know, there are no previous reports on GR and IR evaluating red flesh dragon fruit. The results of the current study for individuals at risk of T2D showed a non-significant decrease in IR and a pattern with a minimal fluctuation in the group receiving the FRDF beverage when compared to the general health advice group. According to previous studies assessing polyphenol-rich food that found a reduction in IR in subjects at risk of T2D, polyphenols may improve insulin sensitivity, probably due to the improvement in insulin signalling in insulin-sensitive tissues [38,39].

Systolic and diastolic pressure showed a decrease (~4 mm Hg units) in individuals at risk of T2D over time in those who consumed the FRDF product. Fruit intake is encouraged as part of a healthy diet, due to their bioactive compounds and their association with reducing the risk of developing chronic non-communicable diseases including cardiovascular disease and T2D [40]. A decrease in blood pressure has been reported in short-term studies in which the effects of the consumption of fresh red dragon fruit on blood pressure have been investigated in healthy participants and individuals who are overweight or obese [12]. The intake of dehydrated (freeze-dried) red flesh dragon fruit resulted in a decrease in both systolic and diastolic blood pressure when compared to a placebo in healthy individuals; however, there was no significant difference between treatments, despite positive changes in endothelial function and arterial stiffness [16]. Nevertheless, the presence of polyphenolic compounds and vitamins E and C found in red dragon fruit in addition to the presence of tocotrienol, a compound related to the cholesterol pathway, has been linked to the positive effects of red flesh dragon fruit consumption on blood pressure [12,41,42].

To our knowledge, this is the first study investigating the effect of FRDF on the lipid profile and biomarkers of inflammation. The results for total cholesterol levels showed a numerical non-significant reduction after two weeks of intervention in subjects at risk of T2D receiving the FRDF beverage. Similarly, Maharani and Saktiningsih [14] evaluated the consumption of red dragon fruit juice in 18 healthy women between 30 and 49 years old, and the results showed a significant cholesterol-lowering effect after 14 days. According to the authors, these findings could be due to the content of antioxidants and fibre in red dragon fruit; furthermore, they linked the reduction in cholesterol to the presence of tocotrienol and anthocyanins, molecules involved in cholesterol metabolism, suppressing synthesis or reducing its production, respectively [14,43]. In this study, triglyceride and CRP levels showed a downward trend after the four-week intervention with the FRDF beverage in individuals at risk of T2D but remained within their normal range (<1.7 mmol/L for triglycerides and <3.0 mg/L for CRP). The effects of polyphenols on CRP levels have been linked to the food matrix, as grapes, raisins, and grape juice showed a significant decrease in CRP levels; meanwhile, grape powder did not, as shown in a meta-analysis performed by Sarkhosh-Khorasani and Hosseinzadeh [44]. Furthermore, the authors stated that the effects of polyphenol on CRP levels are mainly a result of the dose and the period of consuming polyphenol-rich food; however, the type of preservation method and storage conditions may influence the positive effects of polyphenol. More than one mechanism has been proposed for the anti-inflammatory effects of polyphenols, such as gene expression and an increase in the intestinal microbiota, which reduce the production of CRP [45,46]. Polyphenols have shown the capacity to inhibit different enzymes involved in oxidative stress such as NADPH oxidase and the ability to enhance antioxidant enzymes such as superoxide dismutase as part of their biological activity, and these mechanisms have been related to their positive anti-inflammatory effects [47]. Based on the above, further research is needed to compare dragon fruit matrix effects on biomarkers.

This study was limited by factors including the following: the period of recruitment took place shortly after security measures were lifted due to the COVID-19 pandemic; the small sample size, due to the lack of participation interest and failure to obtain consent from many participants to having venipuncture; also there were budget and time constraints. Therefore, further research is warranted including studies with a larger, full-powered sample size and for a longer period of treatment; studies using different methods for increasing recruitment, either emphasizing an incentive to be obtained at the end of the trial or the contribution that the results of the study will provide to the health of the population are assured. Furthermore, the consumption of exotic fruits is not common in the community where this study was established, so conducting a trial in a setting where participants are familiar with the fruit is highly recommended to reduce the number of drop outs. Moreover, variations in the preparation of the beverage at home might have influenced the results, and finally, another limitation could be the possible slight variations in polyphenol content between the batches of the fruit and the contribution of polyphenols from the habitual diet of participants. Despite the randomization of participants to the FRDF group and general health advice group, there was no intentional stratification of volunteers into each group based on age, gender, or other baseline variables. This may have contributed to the variation in some results noted within each group. Moreover, the aim of this study was to test a natural dragon fruit-based drink with minimal processing, which led to practical difficulties in including a placebo beverage to conduct a double-blind intervention.

## 5. Conclusions

In conclusion, an FRDF-based beverage may have the potential to reduce blood pressure and IR-iAUC in individuals at risk of T2D, in addition to improving the lipid profile and CRP levels in this population. However, due to the limitations of this study, further research is needed to elucidate the mechanisms involved in reducing these metabolic outcomes and the influence of individual polyphenols contained in FRDF to further understand their role in health outcomes.

## Figures and Tables

**Table 1:** The characteristics of the participants.

	At Risk of T2D	Healthy
Participants	9 (7F, 2M) (2A, 2B, 1Mi, 3W, 1O)	9 (5F, 4M) (3A, 6W)
Age (years)	31.7 ± 12.1	28.4 ± 5.2
Height (cm)	168.7 ± 9.1	169.7 ± 5.3
Weight (kg)		
Pre-intervention	82.6 ± 16.6 *	62.4 ± 13.0 *
Post-intervention	82.9 ± 16.4 *	65.2 ± 8.4 *
BMI (kg/m^2^)		
Pre-intervention	29.0 ± 5.6 *	22.7 ± 1.9 *
Post-intervention	29.1 ± 5.4 *	22.2 ± 1.7 *

cm: centimetres; kg: kilograms; BMI: body mass index; kg/m^2^: kilograms per square metre; T2D: type 2 diabetes; F: female; M: male; A: Asian; B: Black; Mi: Mixed; W: White; O: Other. Values are presented as mean ± standard deviation. Asterisk (*) denotes that mean values in same row were significantly different at *p* < 0.05.

**Table 2:** Fasting blood glucose and 2 h glucose concentration after consuming standard glucose drink in groups that received general health advice or FRDF-based beverage at week-0, week-2, and week-4 after treatment.

	General Health Advice		FRDF-Based Beverage	
Blood Glucose (mmol/L)	At Risk of T2D	Healthy	At Risk of T2D	Healthy
Fasting				
Week-0	4.8 ± 0.9	4.3 ± 0.4 *	5.1 ± 0.5	4.8 ± 0.6 *
Week-2	4.6 ± 0.4	4.3 ± 0.3 *	5.1 ± 0.3	5.1 ± 0.5 *
Week-4	4.9 ± 0.8	4.1 ± 0.4 *	5.2 ± 0.1	4.8 ± 0.4 *
2 h after				
Week-0	5.2 ± 1.1 *	5.2 ± 0.6	7.3 ± 0.9 *	5.2 ± 1.1
Week-2	5.6 ± 0.7 *	4.7 ± 0.5	6.3 ± 0.7 *	5.9 ± 1.6
Week-4	5.2 ± 1.4 *	4.7 ± 1.4	6.6 ± 0.6 *	4.9 ± 0.8

FRDF: frozen red dragon fruit; mmol/L: millimol per litre; T2D: type 2 diabetes. Values represented as mean ± standard deviation. (*n* = 18). Asterisk (*) in same row denotes significant difference between treatments at *p* < 0.05.

**Table 3:** iAUC for blood glucose for groups that received general health advice or red flesh dragon fruit-based beverage at week-0, week-2, and week-4 after treatment.

	General Health Advice		FRDF-Based Beverage	
iAUC	At Risk of T2D	Healthy	At Risk of T2D	Healthy
Glucose				
Week-0	140.5 ± 80.0 *	182.3 ± 81.4	313.8 ± 43.1 *	175.6 ± 87.9
Week-2	192.7 ± 92.6 *	189.2 ± 147.7	301.6 ± 132.2 *	211.4 ± 122.8
Week-4	168.5 ± 62.6 *	156.6 ± 72.9	314.0 ± 124.8 *	188.0 ± 106.9

iAUC: incremental area under the curve; T2D: type 2 diabetes. Values are presented as mean ± standard deviation. Asterisk (*) in same row denotes significant difference between treatments at *p* < 0.05.

**Table 4:** iAUC for plasma insulin for groups that received general health advice or red flesh dragon fruit-based beverage at week-0, week-2, and week-4 after treatment.

	General Health Advice		FRDF-Based Beverage	
iAUC	At Risk of T2D	Healthy	At Risk of T2D	Healthy
Insulin				
Week-0	8486.7 ± 10,230.5	5387.5 ± 2383.0	7777.2 ± 6004.9	5457.2 ± 5102.9
Week-2	11,135.4 ± 9249.9	4238.1 ± 1452.6	7292.3 ± 3880.0	8361.5 ± 10,758.7
Week-4	10,798.7 ± 11,489.9	4044.0 ± 3356.4	5311.8 ± 4400.4	4001.2 ± 984.9

iAUC: incremental area under the curve; T2D: type 2 diabetes. Values are presented as mean ± standard deviation.

**Table 5:** Blood pressure for groups that received general health advice or FRDF-based beverage at week-0, week-2, and week-4 after treatment.

	General Health Advice		FRDF-Based Beverage	
Blood Pressure (mm Hg)	At Risk of T2D	Healthy	At Risk of T2D	Healthy
Systolic				
Week-0	110 ± 6	108 ± 10	113 ± 4	104 ± 14
Week-2	110 ± 8	103 ± 5	111 ± 5	102 ± 12
Week-4	110 ± 10	108 ± 6	109 ± 8	104 ± 9
Diastolic				
Week-0	79 ± 9	67 ± 6	81 ± 8	69 ± 6
Week-2	77 ± 11	65 ± 4	80 ± 2	67 ± 7
Week-4	77 ± 14	68 ± 5	77 ± 11	68 ± 2

FRDF: frozen red dragon fruit; mm Hg: millimetres of mercury; T2D: type 2 diabetes. Values represented as mean ± standard deviation (*n* = 18).

**Table 6:** Biomarkers in serum for groups that received general health advice or FRDF-based beverage at week-0, week-2, and week-4 after treatment.

	General Health Advice		FRDF-Based Beverage	
Biomarkers	At Risk of T2D	Healthy	At Risk of T2D	Healthy
Cholesterol (mmol/L)				
Week-0	0.61 ± 0.08	0.54 ± 0.02	0.72 ± 0.59	ND
Week-2	0.62 ± 0.05	0.52 ± 0.00	0.66 ± 0.08	ND
Week-4	0.68 ± 0.03	0.52 ± 0.04	0.71 ± 0.20	ND
Triglycerides (mmol/L)				
Week-0	0.78 ± 0.29	0.24 ± 0.07	0.37 ± 0.27	ND
Week-2	0.57 ± 0.22	0.34 ± 0.11	0.48 ± 0.37	ND
Week-4	0.68 ± 0.16	0.33 ± 0.18	0.29 ± 0.15	ND
TAS (mmol/L)				
Week-0	0.28 ± 0.01	0.38 ± 0.05	0.28± 0.02	ND
Week-2	0.29 ± 0.02	0.37 ± 0.06	0.31 ± 0.07	ND
Week-4	0.30 ± 0.04	MD	0.31 ± 0.08	ND
CRP (mg/L)				
Week-0	10.23 ± 5.92 *	0.41 ± 0.00	0.83 ± 0.45 *	ND
Week-2	8.83 ± 2.02 *	0.60 ± 0.18	0.58 ± 0.22 *	ND
Week-4	12.54 ± 12.98 *	0.45 ± 0.05	0.53 ± 0.20 *	ND

T2D: type 2 diabetes; FRDF: frozen red dragon fruit; mmol/L: millimol per litre; ND: no data; TAS: total antioxidant status; CRP: C-reactive protein; mg/L: milligrams per litre; MD: missed data. Values are presented as mean ± standard deviation. Mean values in same row with asterisk (*) were significantly different at *p* < 0.05.

## Data Availability

The data presented in this study are unavailable due to privacy or ethical restrictions.
